# Backward illusory line motion: Visual motion perception can be influenced by retrospective stimulation

**DOI:** 10.1167/jov.23.6.6

**Published:** 2023-06-12

**Authors:** Fuminori Ono, Yuki Yamada, Kohske Takahashi, Kyoshiro Sasaki, Atsunori Ariga

**Affiliations:** 1 Department of Education, Yamaguchi University, Yamaguchi, Japan; 2 Faculty of Arts and Science, Kyushu University, Fukuoka, Japan; 3 College of Comprehensive Psychology, Ritsumeikan University, Osaka, Japan; 4 Faculty of Informatics Department of Informatics, Kansai University, Osaka, Japan; 5 Faculty of Letters, Chuo University, Tokyo, Japan

**Keywords:** illusory line motion, attention, postdiction, motion perception, stimulus motion

## Abstract

When a visual cue appears beside a horizontal line segment before the line appears, the illusory motion is perceived as a line extending from the side closest to the side farthest from the cue. This is known as illusory line motion (ILM). In Experiment 1, we presented the cue after the line onset and found that the line seemed to extend toward the side of the cue (backward ILM). In Experiment 2, we confirmed the robustness and replicability of the backward ILM. In Experiments 3 to 5, we investigated the role of endogenous and exogenous attention in the generation of backward ILM and found effects of attention, but not large enough to explain the backward ILM in Experiments 1 and 2. The current findings suggest that the direction of ILM depended on the temporal relation of whether the cue precedes or follows the stimulus appearance, and that attentional shift played a role in the perception of backward ILM.

## Introduction

Attention is believed to serve as a filter in processing sensory input information, facilitating processing where attention is directed and inhibiting processing where attention is not (Broadbent, 1958). For example, attention improves various fundamental aspects of visual processing, such as increased spatial and temporal resolution, faster responses, and more accurate reactions (Carrasco, Williams, & Yeshurun, 2002; Posner, 1980; Posner & Petersen, 1990; Yeshurun, 2004; Yeshurun & Carrasco, 1998, 1999). Attention also modifies apparent aspects of visual stimuli, such as apparent spatial frequency, contrast, gap size, texture segmentation, and spatial configuration (Carrasco, Ling, & Read, 2004; Gobell & Carrasco, 2005; Pratt & Arnott, 2008; Suzuki & Cavanagh, 1997; Yeshurun, Montagna, & Carrasco, 2008). These findings indicate that attention acts before or during the presentation of the visual stimulus.

There are several perceptual phenomena in which a stimulus presented later appears to influence the perception of another stimulus presented earlier (Shimojo, 2014). In backward masking, a classic example of retrospective phenomena, if a visual stimulus is presented immediately after another visual stimulus is presented in the same location, the previously presented stimulus may become invisible (Breitmeyer & Öğmen, 2006). Other such retrospective phenomena include the color phi phenomenon (Kolers & von Grünau, 1975, 1976), the flash-lag effect (Eagleman & Sejnowski, 2000), the chronostasis effect (Yarrow, Haggard, Heal, Brown, & Rothwell, 2001), the attentional attraction effect (Ono & Watanabe, 2011), and the causal capture effect (Choi & Scholl, 2006). We investigated whether visual attention after stimulus presentation alters the perception of that stimulus using the phenomenon of illusory line motion (ILM).

When a visual spatial cue is presented near either end of a line segment before the line is presented, the line seems to extend from the cue side, even though the whole line is drawn simultaneously. This perception of apparent motion is generally referred to as ILM. Hikosaka, Miyauchi, and Shimojo (1993a) proposed an explanation for ILM based on the facilitating effect of spatial attention. In this explanation, the presentation of the cue attracts the observer's attention to that location. Since information processing at the location toward which attention is directed is accelerated, compared to other locations (Stelmach & Herdman, 1991), the processing of the line is prioritized from the cue side. Consequently, there is a gradient in the line's processing speed, and the line is perceived as if it appeared to extend from the cue side. The results of several studies also support the idea that ILM is caused by spatial attention. For example, ILM and the effects of spatial attention reportedly exhibit similar temporal transitions (Hikosaka, Miyauchi, & Shimojo, 1993b); that is, ILM is produced when observers intentionally direct their attention to a certain location in the visual field (Hikosaka et al., 1993b; Schmidt, 2000) and when observers’ attention is guided by another person's gaze toward a particular direction (gaze cueing) (Bavelier, Schneider, & Monacelli, 2002). Furthermore, ILM is also shown to occur when auditory stimuli and tactile vibrations are used as cue stimuli (Shimojo, Miyauchi, & Hikosaka, 1997), indicating that ILM is independent of the modality of the cue. These results support the idea that the ILM is caused by the spatial attention attracted by the cue presented before the line (i.e., pre-cue), regardless of attention-attracting methods or stimuli.

We investigated the time span of the cue that makes attention effective, focusing on both pre-cue and post-cue. We defined the cue presented before the line as the pre-cue and the cue presented after the line as the post-cue. Using ILM as an index of the spatiotemporal characteristics of attention is an efficient method to understand how attention affects the automatic cascade of visual processing against time flow. By manipulating stimulus onset asynchrony (SOA) between the cue and line presentation, Hikosaka et al. (1993a) demonstrated that ILM effects peaked when the cue was presented approximately 200 ms before the presentation of the line. In this study, the SOA between the cue and the line was also manipulated; however, the SOA was extended to the time after the onset of the line. Shimojo et al. (1997) reported that two out six participants predominantly perceived the line motion toward the cue side when a cue was presented after the line. Shimojo et al. (1997) attributed this to backward masking, which decreases the visibility of the line segment near the cue. We re-examined the effect of post-cue in Experiments 3 through 5, eliminating the effect of backward masking.

## Experiment 1

In Experiment 1, the SOA between the line and cue presentation was set at greater intervals, including after the presentation of the line; the change in perception of ILM owing to the difference in SOA was examined. Hikosaka et al. (1993a) demonstrated obvious changes in illusion strength based on cue-line SOA. Therefore, we hypothesized that this would also occur in the post-cue conditions used here.

### Methods

#### Participants

We determined a required sample size based on a power analysis. Since the effect size and other statistical values were not reported specifically in Shimojo et al. (1997), we adopted a middle effect size, Cohen's *f* = 0.3, for the power analysis. We calculated the sample size required for detecting a significant main effect in a two-way analysis of variance (ANOVA) using G*power, Cohen's *f* = 0.3, *α* = 0.05, 1 – *β* = 0.95 (Faul, Erdfelder, Lang, & Buchner, 2007; Faul, Erdfelder, Buchner, & Lang, 2009). The required sample size was set at 14, and 14 participants (6 women and 8 men), aged between 20 and 24 years, were recruited for the experiment. All participants had normal or corrected vision. The ethics committee of Yamaguchi University (no. 2018-001-02) approved this study. The experiment was conducted according to the guidelines of the Helsinki Declaration. We obtained written informed consent from all participants before the experiment.

#### Apparatus and stimuli

Participants viewed the stimuli on an LCD monitor (Dell AW2521HFL, refresh rate 120 Hz) from approximately 60 cm, in a dimly lit, quiet room. Experimental stimuli used in this study were programmed in MATLAB using the Psychophysics Toolbox extensions (Brainard, 1997; Pelli, 1997). All stimuli were presented on a black background on the screen. The initial display consisted of a white fixation cross of 0.3° diameter in the center of the screen. The placeholders were two white 0.3° squares, located 1° above and 1.8° to the left and right of the center of the screen. The cue was a red circle with a diameter of 0.8°, located 1.4° to the left or right of the edge of the line segment. The line was a white 3.6° long and 0.3° thick line positioned between the placeholders. The line was presented in three patterns. The first was presented such that it extends from left to right (actual line motion): Specifically, a line segment of one-third of the total length was presented at a tangent to the left placeholder, a line segment of one-third of the total length was added in the center after 8 ms, and a line segment of the remaining one-third of the total length was added after another 8 ms. The second was presented in the opposite order of presentation as the first, extending from right to left. In the third, the entire line segment was presented at once.

#### Procedure

**Figure 1.**
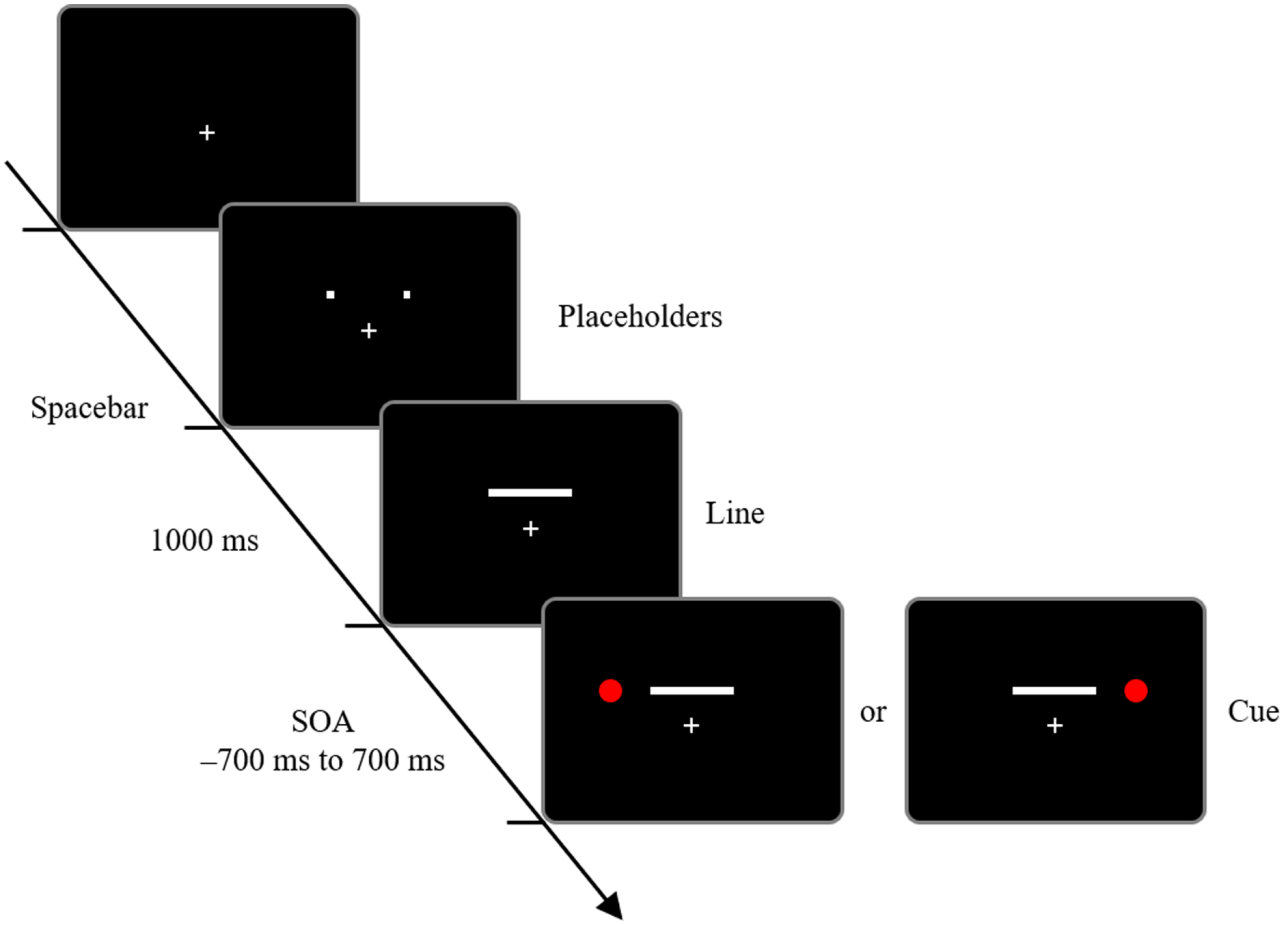
Timeline of a sample trial in Experiment 1. After placeholders (1 second), a cue (circle) and a line were presented. In some trials, the line appeared first (shown here), and in other trials, the cue appeared first. SOA, stimulus onset asynchrony between the cue and line presentation.

Figure 1 demonstrates the timeline of the trial. Participants initiated each trial by tapping on the spacebar. Following this, the placeholders were presented. After 1 s of the appearance of the placeholders, the cue or line was presented. The cue-line SOA was chosen randomly from 13 intervals (−700, −400, −200, –100, −50, −16, 0, 16, 50, 100, 200, 400, or 700 ms). A negative SOA indicated that the cue was presented before the line, and a positive SOA indicated that the cue was presented after the line. Participants judged whether the line seemed to extend from left to right or from right to left and responded by pressing the key corresponding with each judgment, and the stimuli disappeared when they responded.

There were 2 cue location conditions (left or right), 13 SOA conditions, 3 line motion directions (left to right, right to left, or simultaneous), and 6 repetitions. Each participant performed 468 trials. The trial order was randomized across participants. Participants took a break after 234 trials. Additionally, participants were allowed to take short breaks anytime they wanted to. Before the experiment, participants performed 10 practice trials.

### Results

**Figure 2.**
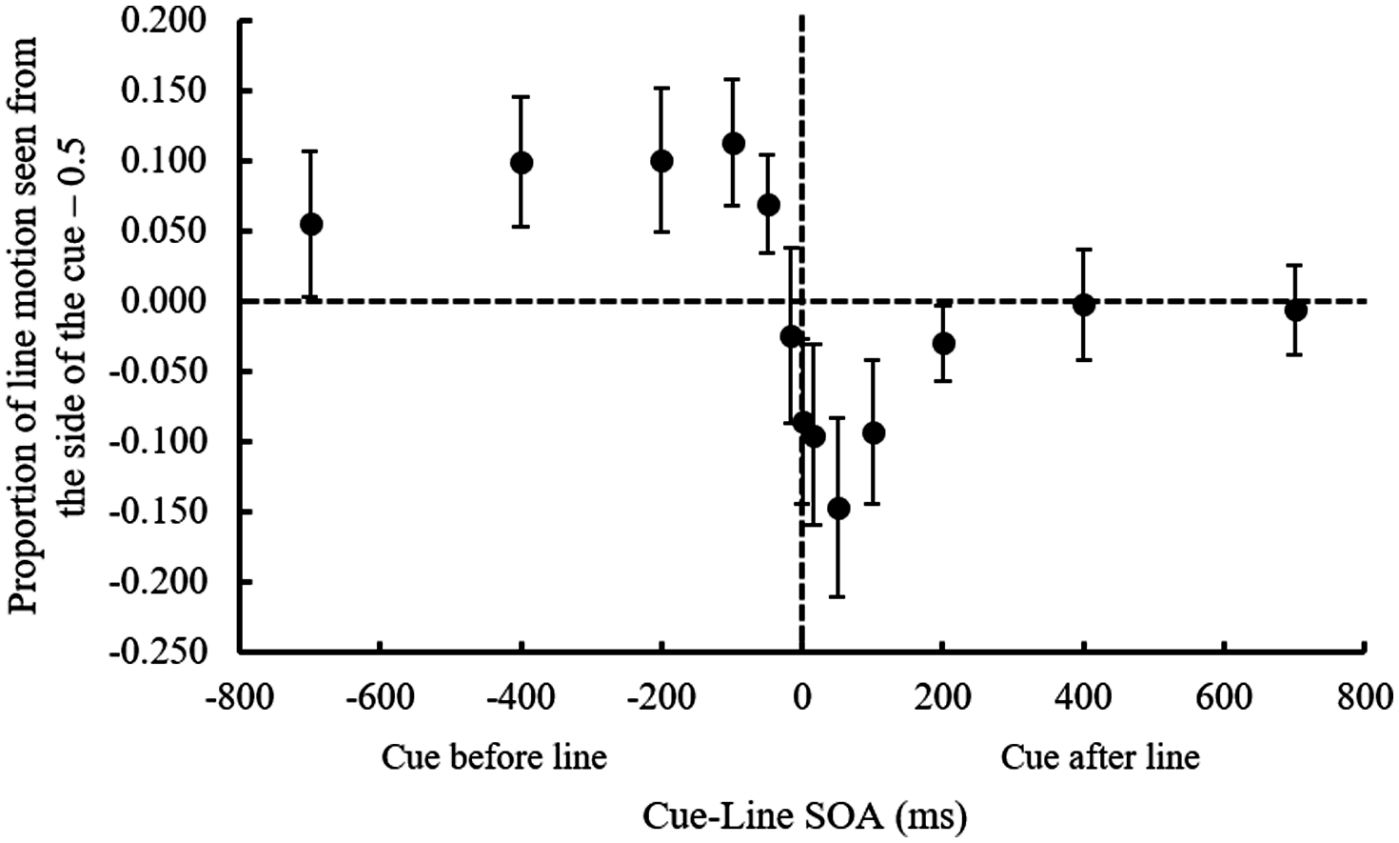
The proportion of illusory line motion perceived at each SOA between the cue and line in Experiment 1. Positive values on the *y*-axis indicate that line motion was perceived from the side of the cue and negative values indicate that it was toward the cue side. Error bars indicate 95% confidence intervals. SOA, stimulus onset asynchrony between the cue and line presentation.

Responses to three line motion directions (left to right, right to left, or simultaneous) were averaged. The outcome variable was the proportion of ILM for each SOA in each cue condition (Figure 2). The ILM was calculated as the mean of the proportion of line motion observed from the side of the cue minus 0.5. A two-way ANOVA with two within-subject variables on the outcome variable showed no significant main effects of the cue location, *F*(1, 13) = 0.051, *p* = 0.825, *η_p_*^2^ = 0.004, and SOA, *F*(12, 156) = 1.653, *p* = 0.107, *η_p_*^2^ = 0.113; however, their interaction was significant, *F*(12, 156) = 21.816, *p* < 0.001, *η_p_*^2^ = 0.627. A simple main effect analysis revealed that the proportion of left-to-right ILM was significantly higher for the left cue than the right cue in the −700 ms, *F(*1, 169) = 5.787, *p* = 0.017, *η_p_*^2^ = 0.308; −400 ms, *F*(1, 169) = 18.453, *p* < 0.001, *η_p_*^2^ = 0.587; –200 ms, *F*(1, 169) = 19.199, *p* < 0.001, *η_p_*^2^ = 0.596; −100 ms, *F*(1, 169) = 23.982, *p* < 0.001, *η_p_*^2^ = 0.648; and –50 ms, *F*(1, 169) = 9.042, *p* < 0.001, *η_p_*^2^ = 0.410, SOA conditions, and that the proportion of right-to-left ILM was significantly higher for the left cue than the right cue in the 0 ms, *F*(1, 169) = 13.648, *p* < 0.001, *η_p_*^2^ = 0.512; 16 ms, *F*(1, 169) = 17.006, *p* < 0.001, *η_p_*^2^ = 0.567; 50 ms, *F*(1, 169) = 40.419, *p* < 0.001, *η_p_*^2^ = 0.757; and 100 ms, *F*(1, 169) = 16.305, *p* < 0.001, *η_p_*^2^ = 0.556, SOA conditions. Thus, if the cue was presented before the line was presented, the line was perceived as extending from the cue side. This ILM effect reached its peak at approximately –100 ms SOA. This result was roughly consistent with the findings of Hikosaka et al. (1993a). In contrast, if the cue was presented after the line was presented, the line was perceived as extending toward the cue side. This opposite direction ILM effect reached its peak at approximately 50 ms SOA.

## Experiment 2

In Experiment 2, we examined the robustness and replicability of the ILM and the opposite direction ILM observed in Experiment 1, using the peak of SOA (–100 ms and 50 ms) in which the respective ILMs were observed. In Experiments 2, 3, and 5, we preregistered our experimental protocols (Experiment 2: https://doi.org/10.17605/OSF.IO/RUKMV; Experiment 3: https://doi.org/10.17605/OSF.IO/TEXNF; Experiment 5: https://doi.org/10.17605/OSF.IO/QT5NM).

### Methods

#### Participants

Because the results of Experiment 1 revealed large effect sizes at –100 ms, *η_p_*^2^ = 0.648, and 50 ms, *η_p_*^2^ = 0.757, our goal was to obtain 0.95 power to detect a large effect size (Cohen's *dz*) of 0.8 for the standard 0.05 alpha error probability (statistical test: one-sample *t* test). We concluded that the required sample size was 19. Nineteen participants (12 women and 7 men), aged between 18 and 24 years, were selected for this experiment. We obtained written informed consent from all participants before the experiment.

#### Apparatus and stimuli

The apparatus and stimuli were the same as in Experiment 1.

#### Procedure

The trial sequence was the same as in Experiment 1, except that the cue line SOA was chosen randomly from two intervals. There were 2 cue location conditions (left or right), 2 SOA conditions (−100 or 50 ms), 3 line motion directions (left to right, right to left, or simultaneous), and 10 repetitions. Each participant performed 120 trials. Before the experiment, participants performed 10 practice trials.

### Results

**Figure 3.**
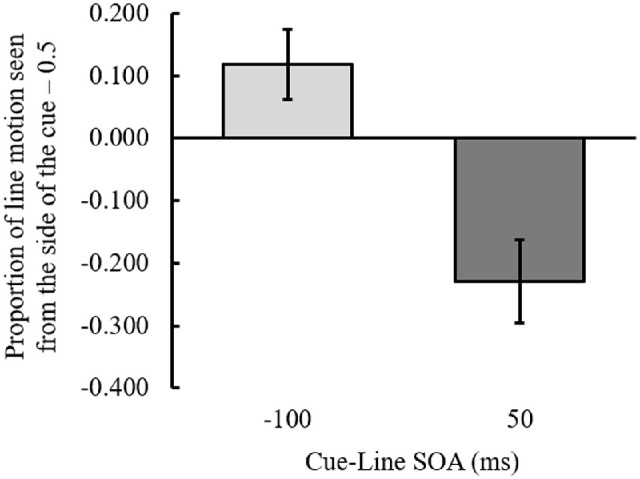
The proportion of illusory line motion perceived at each SOA between the cue and line in Experiment 2. Positive values on the *y*-axis indicate that line motion was perceived from the cue side, and negative values indicate that it was toward the cue side. Error bars indicate 95% confidence intervals. SOA, stimulus onset asynchrony between the cue and line presentation.

Responses to the three line motion directions (left to right, right to left, or simultaneous) were averaged. The outcome variable was the proportion of ILM perceived at each SOA (Figure 3); the ILM was calculated as the mean of the proportion of line motion observed from the side of the cue minus 0.5. A one-sample *t* test to determine whether the ILM was greater or less than 0 at each SOA was performed, and the results demonstrated that when the SOA was –100 ms, the ILM was significantly greater than 0, *t*(18) = 4.385, *p* < 0.001, Cohen's *dz* = 1.000; conversely, when the SOA was 50 ms, the ILM was significantly smaller than zero, *t*(18) = 7.262, *p* < 0.001, Cohen's *dz* = 1.672. These results additionally supported that ILM was perceived when the cue was presented before the line was presented, and ILM in the opposite direction was perceived when the cue was presented after the line was presented. We refer to the opposite direction ILM produced by the cue after the line onset as backward ILM.

## Experiment 3

In Experiment 3, we tested the hypothesis that backward ILM can be influenced by endogenous attention. To rule out factors other than endogenous attention, left and right cues were presented simultaneously, with a task-related cue and an unrelated cue. If backward ILM can be influenced by endogenous attention, we expected that the location of the task-related cue would determine the direction of the perceived line motion. Therefore, if the cues were presented before the line, ILM was expected to be perceived from the task-related cue side; if the cues were presented after the line, ILM was expected to be perceived toward the task-related cue side.

### Methods

#### Participants

As in Experiment 2, our goal was to obtain 0.95 power to detect a large effect size (Cohen's *dz*) of 0.8 for the standard 0.05 alpha error probability. A one-sample *t* test was conducted. The colors of the cues were red and green, with different colors in the first and second one-half of the experiment that were task related. To counterbalance the task-related color of the cue (i.e., 10 for the red-attended-first group and the remaining 10 for the green-attended-first group), we recruited 1 additional participant from the original required sample size of 19 and set the required sample size at 20. Twenty participants (11 women and 9 men), aged 18 to 24 years, were recruited for the experiment. We obtained written informed consent from all participants before the experiment.

#### Apparatus and stimuli

The apparatus and stimuli were the same as in Experiments 1 and 2, except for the following: The cues were presented for 8 ms on both the left and right sides of the line. In Experiments 1 and 2, the cue was presented until the participant responded; however, in Experiment 3, the cue disappeared 8 ms after presentation. The cue was a circle with a diameter of 0.8° or a square with a 0.7° length on each side, and red or green in color. The shape of the cue was chosen randomly and independently for left and right, but each pair included a red cue and a green cue.

#### Procedure

**Figure 4.**
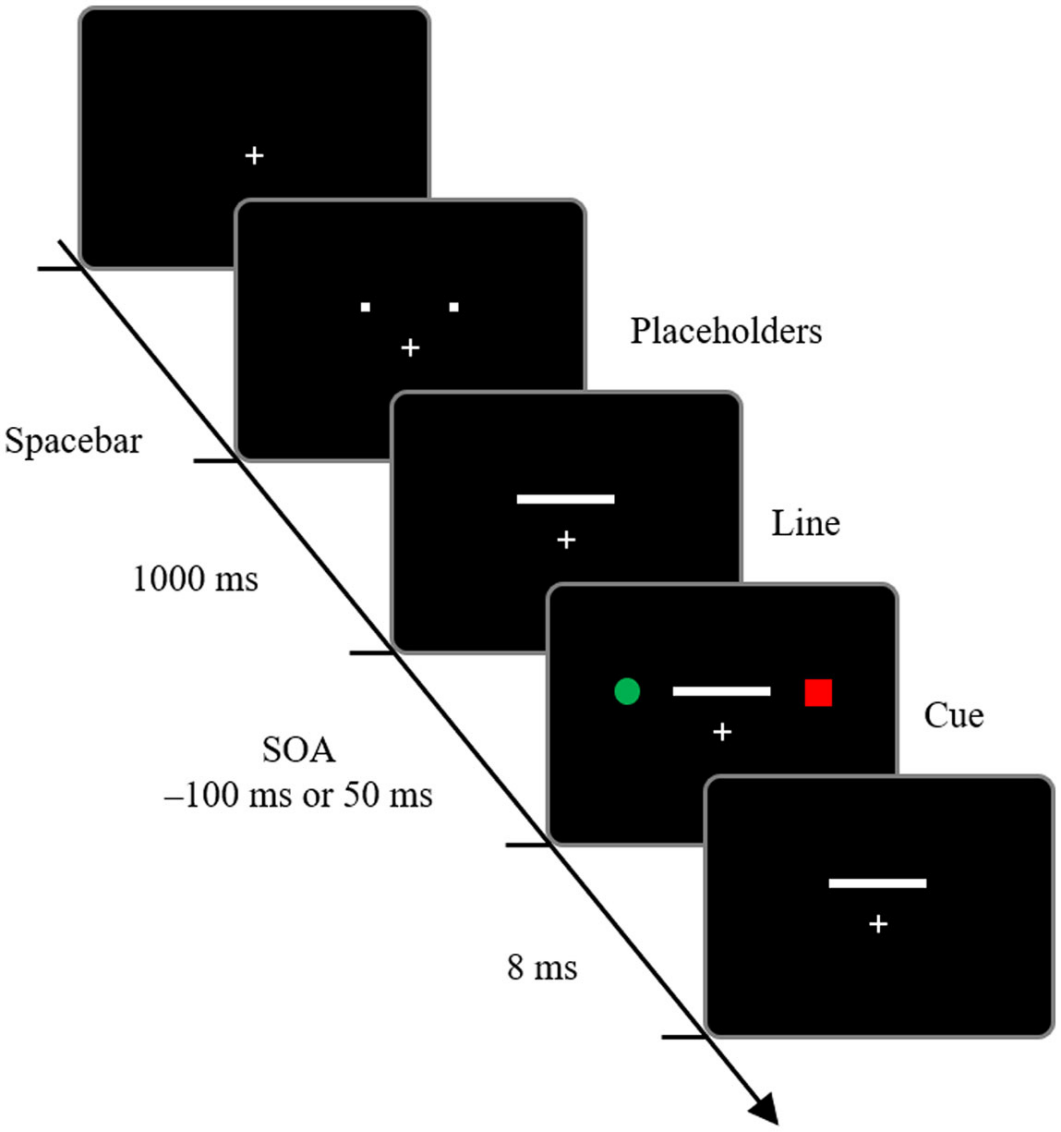
Timeline of a sample trial in Experiment 3. The trial sequence was the same as in Experiments 1 and 2, except that it was a shape judgment task to direct participants’ attention to only one cue. SOA, stimulus onset asynchrony between the cue and line presentation.

Figure 4 demonstrates the timeline of a trial. In Experiment 3, two tasks were set to direct participants’ attention to only one cue. The first task was to judge the direction of line motion, as in Experiments 1 and 2, and the second task was to judge the shape of a particular color cue (shape judgment task). The experiment consisted of two blocks. One-half of the 20 participants were instructed to respond to the shape of the red cue in the first block and the shape of the green cue in the second block; the other one-half were instructed in the opposite order. There were 2 attended-cue location conditions (left or right), 2 SOA conditions (−100 or 50 ms), 3 line motion directions (left to right, right to left, or simultaneous), and 20 repetitions (10 for the first one-half of the block, 10 for the second one-half of the block). Each participant performed 240 trials. Before the experiment, participants performed 10 practice trials.

### Results

**Figure 5.**
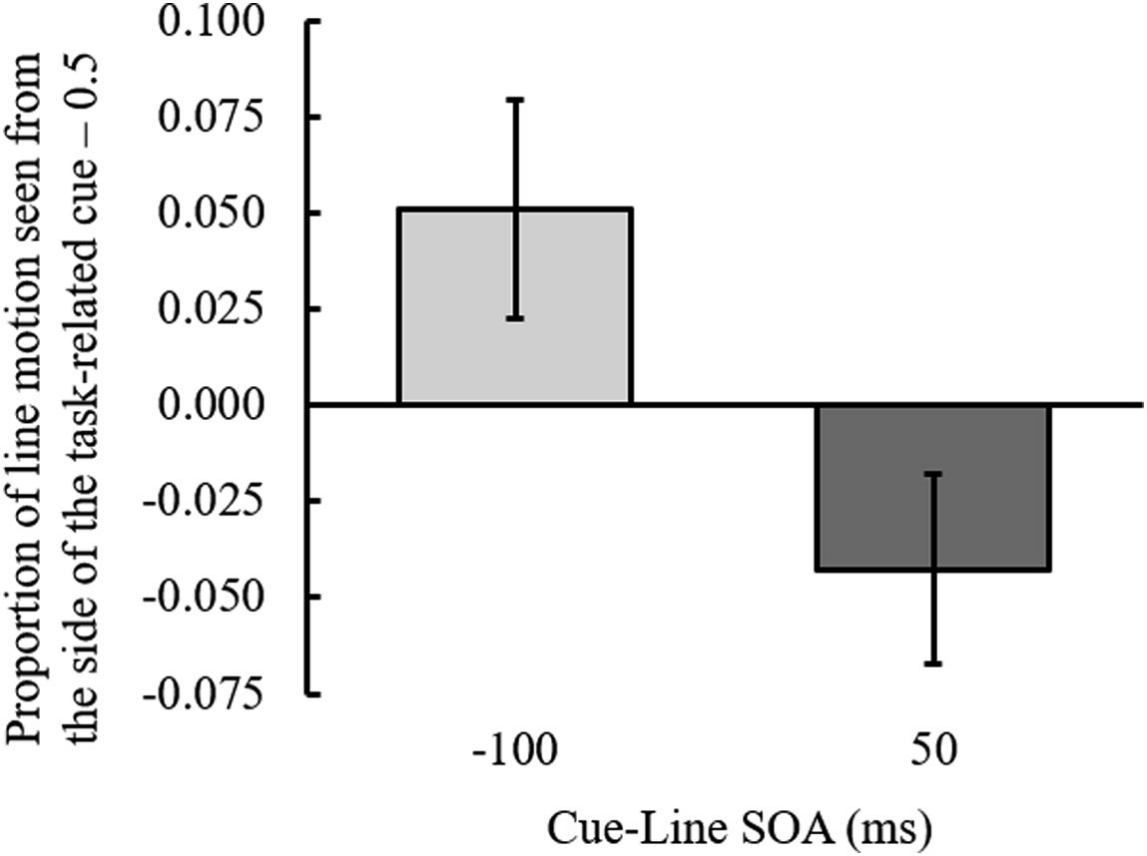
The proportion of ILM perceived at each SOA between the cue and line in Experiment 3. Positive values on the *y*-axis indicate that line motion was perceived from the side of the attentional cue, and negative values indicate that it was toward the side of the task-related cue. Error bars indicate 95% confidence intervals. SOA, stimulus onset asynchrony between the cue and line presentation.

We analyzed data only for the trials in which the shape judgment task was judged correctly. The mean accuracy for the shape judgment task was 96.8%. Responses to three line motion directions (left to right, right to left, or simultaneous) were averaged. The outcome variable was the proportion of ILM perceived at each SOA (Figure 5^6^); the ILM was calculated as the mean of the proportion of line motion observed from the side of the task-related cue minus 0.5. A one-sample *t* test to determine whether the ILM was greater or less than 0 at each SOA was performed, and the results revealed that when the SOA was –100 ms, the ILM was significantly greater than 0, *t*(19) = 3.758, *p* = 0.001, Cohen's *dz* = 0.840; conversely, when the SOA was 50 ms, the ILM was significantly smaller than 0, *t*(19) = 3.612, *p* = 0.002, Cohen's *dz* = 0.808. These results indicate that ILM was perceived when the task-related cue was presented before the line, and backward ILM was perceived when the task-related cue was presented after the line. These findings suggest that endogenous attention has an effect on the generation of ILM and backward ILM.

**Figure 6.**
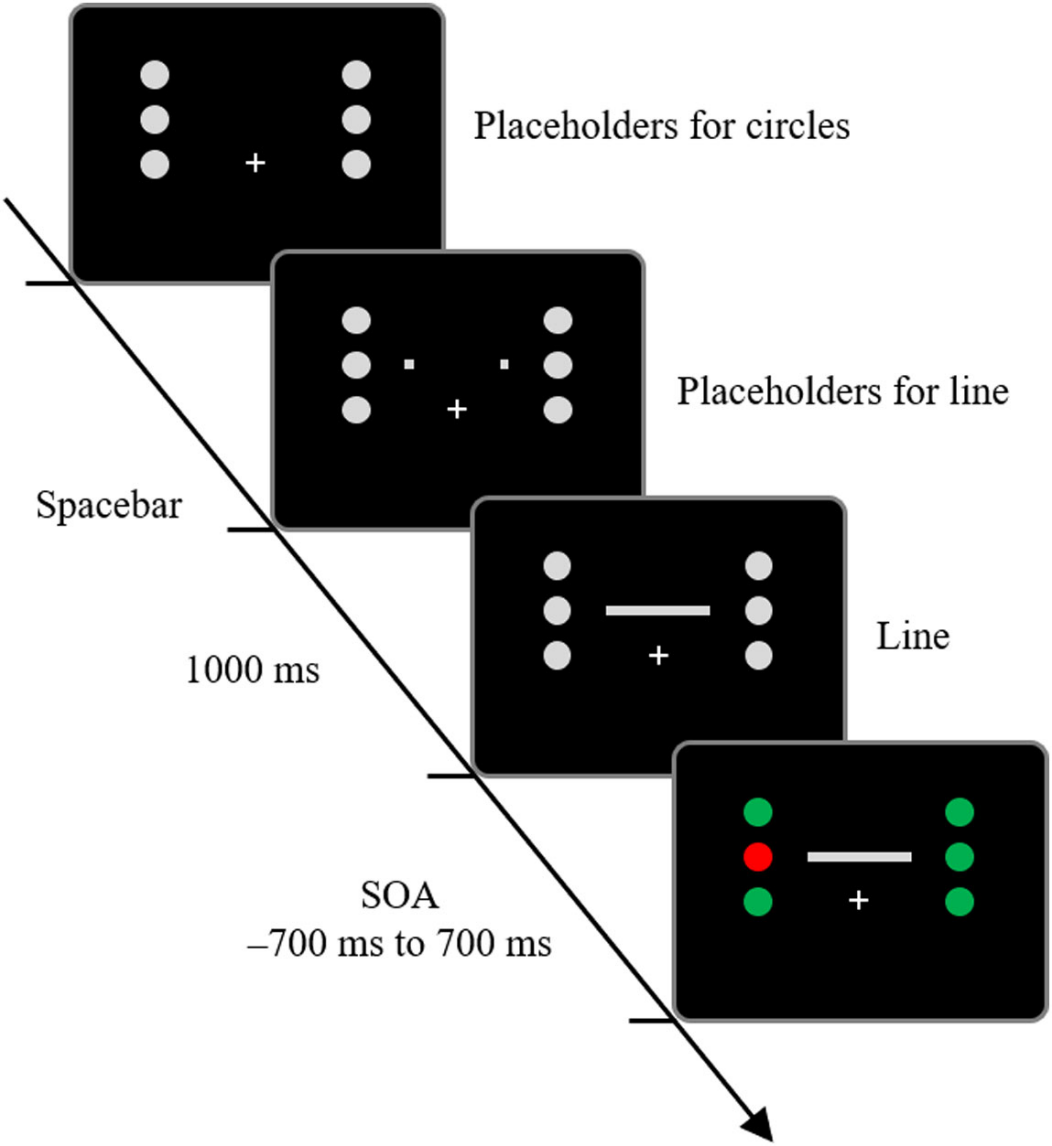
Timeline of a sample trial in Experiment 4. The trial sequence was the same as in Experiments 1 and 2. SOA, stimulus onset asynchrony between the cue and line presentation.

## Experiment 4

In Experiment 4, we tested the hypothesis that backward ILM can be influenced by exogenous attention. To investigate exogenous attentional factors, circles were presented in the periphery alongside the left and right cues. Attention was drawn to only one cue by making one of the left and right cues a different color than the other circles. If backward ILM is influenced by exogenous attention, we expected that the location of the cue attract attention would determine the direction of perceived line motion. Therefore, if the cues were presented before the line, ILM was expected to be perceived from the attention-attracting cue side; if the cues were presented after the line, ILM was expected to be perceived toward the attention-attracting cue side.

### Methods

#### Participants

We adopted a middle effect size, Cohen's *f* = 0.3, for the power analysis. A two-way ANOVA was conducted. We concluded that the required sample size was 14. Fourteen participants (9 women and 5 men), aged between 19 and 23 years, were selected for this experiment. We obtained written informed consent from all participants before the experiment.

#### Apparatus and stimuli

The apparatus and stimuli were the same as in Experiments 1 and 2, except for the following: the cues were presented on both the left and right sides of the line, one with a red circle and the other with a green circle. In addition, a circle of the same diameter as the cue was presented 1.2° above and below the left and right cues. The color of the surrounding four circles was red in the experiment with one-half the participants and green in the experiment with the other one-half. If the surrounding four circles are green in color, the red cue is likely to attract attention, and if the surrounding four circles are red in color, the green cue is likely to attract attention. The color of the line and line placeholders was gray. The placeholders for circles were also presented at the same place as the red and green circles. To eliminate as much as possible the influence of the motion signal caused by the difference in luminance, the luminance of the line segment, the circles, and the placeholders for the line and circle were all kept approximately the same.

#### Procedure

Figure 6 demonstrates the timeline of the trial. The trial sequence was the same as in Experiments 1 and 2. Each participant performed 468 trials. Before the experiment, participants performed 10 practice trials.

### Results

**Figure 7.**
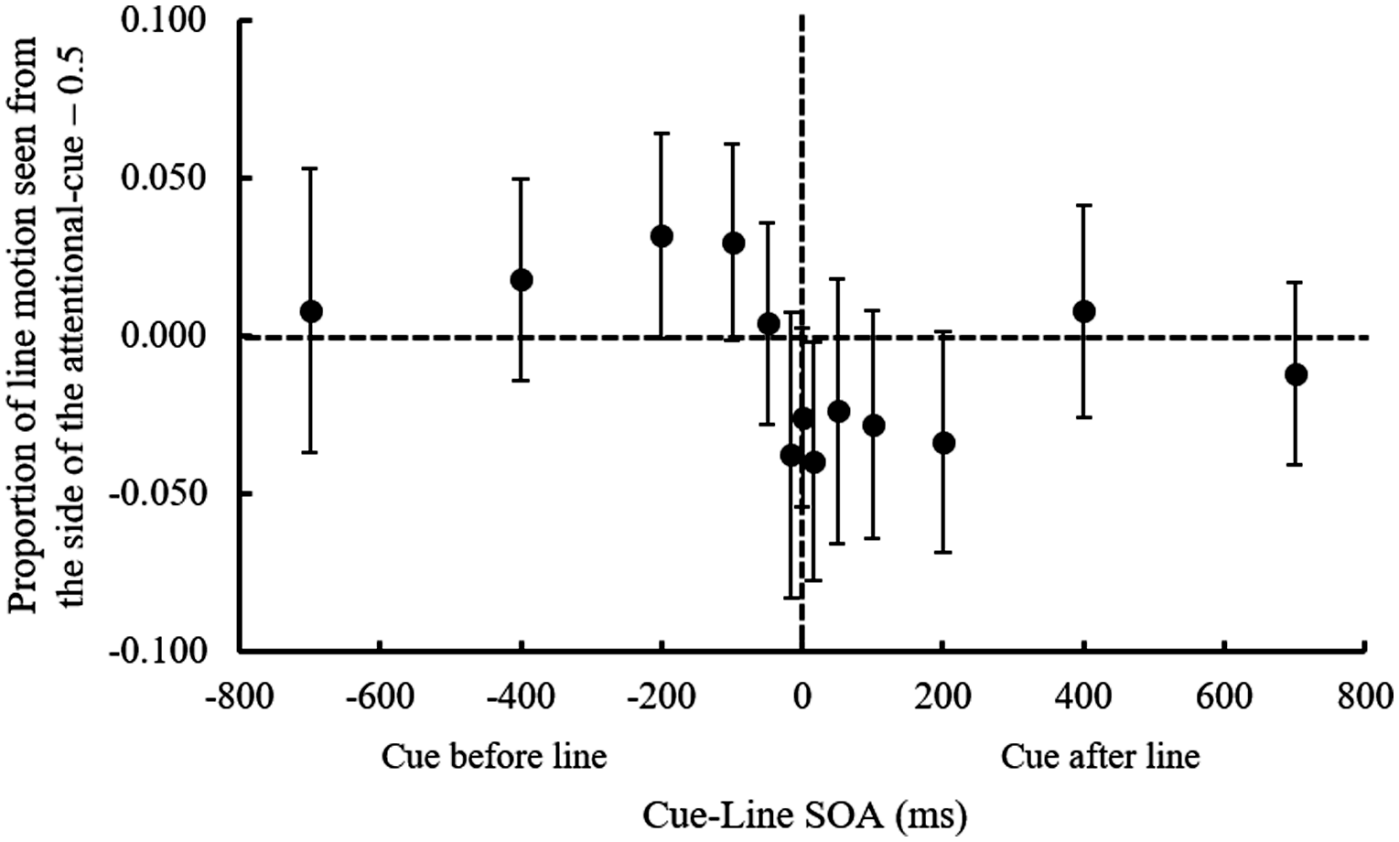
The proportion of ILM perceived at each SOA between the cue and line in Experiment 4. Positive values on the *y*-axis indicate that line motion was perceived from the side of the attentional cue and negative values indicate that it was toward the attentional cue side. Error bars indicate 95% confidence intervals. SOA, stimulus onset asynchrony between the cue and line presentation.

Responses to three line motion directions (left to right, right to left, or simultaneous) were averaged. The outcome variable was the proportion of ILM for each SOA in each cue condition (Figure 7). The ILM was calculated as the mean of the proportion of line motion observed from the side of the cue minus 0.5. A two-way ANOVA with two within-subject variables on the outcome variable showed no significant main effect of the cue location, *F*(1, 13) = 2.001, *p* = 0.181, *η_p_*^2^ = 0.133. The main effect SOA and the interaction were significant, *F*(12, 156) = 2.167, *p* = 0.016, *η_p_*^2^ = 0.143; *F*(12, 156) = 2.476, *p* = 0.005, *η_p_*^2^ = 0.160. A simple main effect analysis revealed that the proportion of right-to-left ILM was significantly higher for the left attentional cue than the right attentional cue in the −16 ms, *F*(1, 169) = 5.186, *p* = 0.024, *η_p_*^2^ = 0.285; 16 ms, *F*(1, 169) = 5.746, *p* = 0.018, *η_p_*^2^ = 0.307; and 200 ms, *F*(1, 169) = 4.151, *p* = 0.043, *η_p_*^2^ = 0.242, SOA conditions. Thus, if the attentional cue was presented after the line was presented, the line was perceived as extending toward the cue side. This backward ILM effect reached its peak at approximately 16 ms SOA.

## Experiment 5

In Experiment 5, we examined the robustness and replicability of the backward ILM induced by exogenous attention observed in Experiment 4, using the peak of SOA (−200 ms and 16 ms).

### Methods

#### Participants

The results of Experiment 4 showed that the effect size (Cohen's *dz*) was 0.609 in the 16 ms SOA condition. Our goal was to obtain 0.95 power to detect the effect size (Cohen's *dz*) of 0.609 at the standard 0.05 alpha error probability. We concluded that the required sample size was 31. Thirty-one participants (19 women and 12 men), aged between 19 and 23 years, were selected for this experiment. We obtained written informed consent from all participants before the experiment.

#### Apparatus and stimuli

The apparatus and stimuli were the same as in Experiment 4. The color of the circles above and below the cues was red with approximately one-half of the participants (16 participants) and green in the experiment with the other one-half (15 participants).

#### Procedure

The trial sequence was the same as in Experiment 4, except that the cue-line SOA was chosen randomly from two intervals. There were 2 cue location conditions (left or right), 2 SOA conditions (−200 or 16 ms), 3 line motion directions (left to right, right to left, or simultaneous), and 10 repetitions. Each participant performed 120 trials. Before the experiment, participants performed 10 practice trials.

### Results

**Figure 8.**
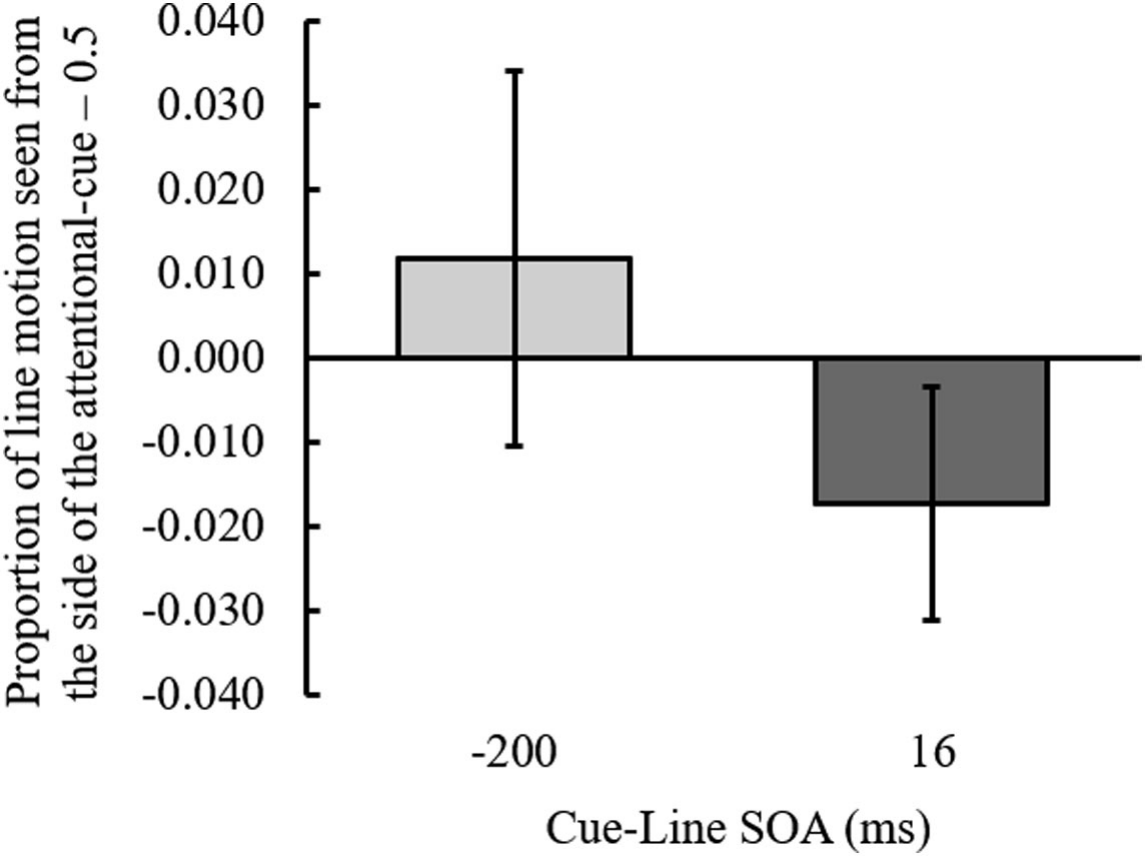
The proportion of ILM perceived at each SOA between the cue and line in Experiment 5. Positive values on the *y*-axis indicate that line motion was perceived from the side of the attentional cue, and negative values indicate that it was toward the side of the attentional cue. Error bars indicate 95% confidence intervals. SOA, stimulus onset asynchrony between the cue and line presentation.

Responses to three line motion directions (left to right, right to left, or simultaneous) were averaged. The outcome variable was the proportion of ILM perceived at each SOA (Figure 8). The ILM was calculated as the mean of the proportion of line motion observed from the side of the attentional cue minus 0.5. A one-sample *t* test to determine whether the ILM was greater or less than 0 at each SOA was performed, and the results demonstrated that when the SOA was –200 ms, the ILM was not significantly different from 0, *t*(30) = 1.084, *p* = 0.287, Cohen's *dz* = 0.195, and when the SOA was 16 ms, the ILM was significantly smaller than zero, *t*(30) = 2.545, *p* = 0.016, Cohen's *dz* = 0.457. The results additionally supported that the backward ILM was perceived when the cue was presented after the line was presented.

## Discussion

The results of these experiments demonstrate that, when a cue is presented after the line segment was presented, the line is perceived as extending toward the cue (backward ILM). This finding is novel for two reasons. First, this study is the first to demonstrate that a cue after the presentation of a visual stimulus induced its motion perception. Second, this perceived motion was in the direction toward the cue. In Experiments 3 through 5, the cues were presented on both the left and right sides of the line. If backward ILM had been perceived solely owing to nonattentional sensory factors, no line motion would have been perceived. Thus, the fact that the attention-attracting post-cue altered the perception of line motion suggests that attention plays a role in eliciting the perception of backward ILM.

We now consider the mechanism of backward ILM. As noted in the Introduction, Shimojo et al. (1997) already reported that some participants (2 out of 6) perceived line motion toward the cue side in an experiment similar to the post-cue condition in this study. They attributed this finding to reduced visibility owing to backward masking. However, in Experiments 3 through 5, the cues were presented on both the left and right sides of the line. If backward masking is caused by the post-cue, then both ends of the line should have been invisible. In addition, although attention was directed to the cue, Boyer and Ro (2007) clarified that the effect of backward masking was attenuated at the location where spatial attention was directed. Therefore, it is unlikely that backward ILM is caused by backward masking.

Regarding the possible mechanism through which ILM is produced, Hikosaka et al. (1993a) proposed that visual input of the line was processed from the side to which attention was directed (the cue side), resulting in the perception that the line appeared to extend from the cue side. However, the results of the post-cue condition in this study indicated that visual input was processed from the opposite side of the cue, which cannot be explained by visual processing of the cue speeding up. In contrast to the view that the mechanism of ILM is based on attentional factors, it has been argued that ILM should be regarded as one of the variations of transformational apparent motion (Downing & Treisman, 1997; Tse, Cavanagh, & Nakayama, 1998). For example, when two stimuli were presented sequentially and the shapes of the first and second stimuli were different, observers could perceive the first stimulus as if the shape of the second stimulus changed smoothly; this is known as impletion (Downing & Treisman, 1997; Farrell & Shepard, 1981). They stated that the ILM could also be explained as part of such an impletion phenomenon. Using a visual search task, Kawahara, Yokosawa, Nishida, and Sato (1996) demonstrated that pre-attentive apparent motion mechanisms may contribute to ILM. However, in the post-cue condition of this study, the first stimulus was the line, and the second stimulus was the line and cue combined; if the impletion phenomenon occurred, the motion would have been perceived with the cue instead of the line. Therefore, the impletion phenomenon cannot explain backward ILM. Eagleman and Sejnowski (2003) reported that motion was perceived in a line segment when a visual stimulus was moved from left to right or right to left of the line, before and after the presentation of the flashed line. This effect was the result of the flashed line being interpreted as a motion streak of the moving visual stimulus, resulting in the perception of line motion. However, because the cues were presented only either before or after the line presentation in this study, the motion streak caused by the cues did not apparently occur. Thus, the current results cannot be explained by motion streak. Downing and Treisman (1997) reported that a line segment appeared to contract in the cue stimulus’ direction when the cue stimulus was presented immediately after the line segment disappeared. Han, Zhu, Corballis, and Hamm (2016) refer to this phenomenon as reverse ILM, and the lack of correlation between reverse ILM and ILM indicates that they are different illusions. However, reverse ILM’s detailed mechanism is unclear.

As described elsewhere in this article, it seems that the previous theories of ILM and other perceptual phenomena cannot explain the mechanism of backward ILM sufficiently. We considered the possibility that backward ILM could be caused by more than one mechanism. Because the strength of ILM and backward ILM seems to be lower in Experiments 3 through 5 than in Experiments 1 and 2, and the peak effect of backward ILM in Experiment 1 was 50 ms, whereas it was 16 ms in Experiment 4, it is possible that the mechanisms involved in Experiments 1 and 2 and Experiments 3 thorugh 5 are not identical. In Experiments 1 and 2, motion (first-order motion) was detected by the luminance flow from the line segment to the cue, which may have led to the perception of motion in the line segment (Cavanagh & Mather, 1989). In Experiments 3 through 5, however, the cue stimuli were presented on the left and right sides of the line segment, which cannot be explained solely by the detection mechanism of first-order motion.

Therefore, we considered the relationship between backward ILM and attention from the perspective of temporal dynamics. Just as the cue attracts the observer's attention, the line is thought to attract attention as well. The pre-cue condition in this study would have resulted in an attentional shift from the cue to the line, while the post-cue condition would have resulted in an attentional shift from the line to the cue. Martinez-Trujillo and Treue (2004) demonstrated that neuronal responses in macaque middle temporal area are modulated by attention. They presented moving random dot patterns to the left and right visual fields and measured cell responses in one receptive field, when attention was directed to the motion pattern in the opposite hemifield. They found that when attention was directed to a motion pattern in the direction the cells preferred (anti-preferred), activity in the receptive field of the opposite hemifield, to which attention was not directed increased (suppressed). Felisberti and Zanker (2005) revealed that in a task in which participants answered whether a particular direction of motion exists from a set of random dots that move smoothly and independently of each other (motion transparency), detection performance improved when the direction of motion to be judged was presented in advance. This result indicates that attention to a specific direction improves human motion detection performance. Based on these findings, attentional shift itself can also serve as a cue for the direction of motion, and the perception of line motion in the post-cue condition may have resulted from a malfunction (over-detection) of the motion detector owing to directional cues by the attentional shift from the line to the cue.

## Conclusions

This study demonstrated that the cue elicits motion perception from the cue side to the subsequent visual stimulus and motion perception toward the cue side to the preceding visual stimulus. Furthermore, it was demonstrated that backward ILM can be caused by both nonattentional sensory factors and attentional shifts. This finding also indicates that attentional shift may play a role in motion perception and possibly in other attention-related phenomena. These phenomena are worth pursuing to further understand the spatiotemporal dynamics of attention.

## Supplementary Material

Supplement 1
