# Supplementary material for: Backward illusory line motion: Visual motion perception can be influenced by retrospective stimulation
Source: J Vis. 2023 Jun 12;23(6):6. doi: 10.1167/jov.23.6.6 (PMC10266553; doi:10.1167/jov.23.6.6)
Supplement: Supplement 1 [file jovi-23-6-6_s001.docx]

Appendix: Analysis of responses to three line-motion directions (left to right, right to left, or simultaneous)


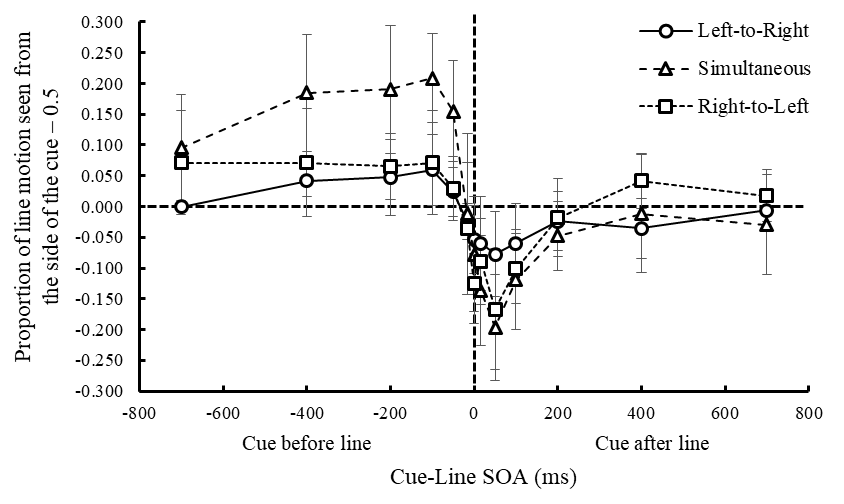


Figure A1. The proportion of illusory line motion (ILM) perceived at each stimulus onset asynchrony (SOA) between the cue and line in each presentation direction of the line in Experiment 1. Positive values on the y-axis indicate that line motion was perceived from the side of the cue and negative values indicate that it was toward the cue side. Error bars indicate 95% confidence intervals.

The responses to line motion in three directions (left to right, simultaneous, or right to left) were analyzed separately. First, responses to left-to-right line motion were included in the analysis. A two-way analysis of variance (ANOVA) with two within-subject variables on the outcome variable showed no significant main effects of the cue location (*F*[1, 13] = 1.632, *p* = .224, *η_p_*^2^ = .112) and SOA (*F*[12, 156] = 1.135, *p* = .335, *η_p_*^2^ = .080); however, their interaction was significant (*F*[12, 156] = 3.664, *p* < .001, *η_p_*^2^ = .220). A simple main effect analysis revealed that the proportion of left-to-right ILM was significantly higher for the left cue than the right cue in the –100 ms (*F*[1, 169] = 5.787, *p* = .017, *η_p_*^2^ = .308) SOA condition, and that the proportion of “right-to-left” ILM was significantly higher for the left cue than the right cue in the 0 ms (*F*[1, 169] = 4.688, *p* = .032, *η_p_*^2^ = .265), 16 ms (*F*[1, 169] = 5.788, *p* = .017, *η_p_*^2^ = .308), 50 ms (*F*[1, 169] = 9.781, *p* = .002, *η_p_*^2^ = .429), and 100 ms (*F*[1, 169] = 5.788, *p* = .017, *η_p_*^2^ = .308) SOA conditions.

Next, responses to no line motion (simultaneous) were included in the analysis. A two-way ANOVA showed no significant main effects of the cue location (*F*[1, 13] = 0.424, *p* = .526, *η_p_*^2^ = .032) and SOA (*F*[12, 156] = 1.099, *p* = .364, *η_p_*^2^ = .078); however, their interaction was significant (*F*[12, 156] = 14.695, *p* < .001, *η_p_*^2^ = .531). A simple main effect analysis revealed that the proportion of left-to-right ILM was significantly higher for the left cue than the right cue in the –700 ms (*F*[1, 169] = 5.194, *p* = .024, *η_p_*^2^ = .285), –400 ms (*F*[1, 169] = 19.497, *p* < .001, *η_p_*^2^ = .600), –200 ms (*F*[1, 169] = 20.775, *p* < .001, *η_p_*^2^ = .615), –100 ms (*F*[1, 169] = 24.853, *p* < .001, *η_p_*^2^ = .657), and –50 ms (*F*[1, 169] = 13.715, *p* < .001, *η_p_*^2^ = .513) SOA conditions, and that the proportion of “right-to-left” ILM was significantly higher for the left cue than the right cue in the 16 ms (*F*[1, 169] = 10.732, *p* = .001, *η_p_*^2^ = .452), 50 ms (*F*[1, 169] = 22.094, *p* < .001, *η_p_*^2^ = .630), and 100 ms (*F*[1, 169] = 8.115, *p* = .005, *η_p_*^2^ = .384) SOA conditions.

Finally, responses to right-to-left line motion were included in the analysis. A two-way ANOVA showed no significant main effects of the cue location (*F*[1, 13] = 0.674, *p* = .429, *η_p_*^2^ = .049). The main effect of SOA (*F*[12, 156] = 2.110, *p* = .019, *η_p_*^2^ = .140) and the interaction were significant (*F*[12, 156] = 7.206, *p* < .001, *η_p_*^2^ = .357). A simple main effect analysis revealed that the proportion of left-to-right ILM was significantly higher for the left cue than the right cue in the –700 ms (*F*[1, 169] = 4.490, *p* = .036, *η_p_*^2^ = .257), –400 ms (*F*[1, 169] = 4.490, *p* = .036, *η_p_*^2^ = .257), and –100 ms (*F*[1, 169] = 4.490, *p* = .036, *η_p_*^2^ = .257) SOA conditions, and that the proportion of “right-to-left” ILM was significantly higher for the left cue than the right cue in the 0 ms (*F*[1, 169] = 13.751, *p* < .001, *η_p_*^2^ = .514), 16 ms (*F*[1, 169] = 7.016, *p* = .009, *η_p_*^2^ = .351), 50 ms (*F*[1, 169] = 24.446, *p* < .001, *η_p_*^2^ = .653), and 100 ms (*F*[1, 169] = 9.011, *p* = .003, *η_p_*^2^ = .409) SOA conditions.


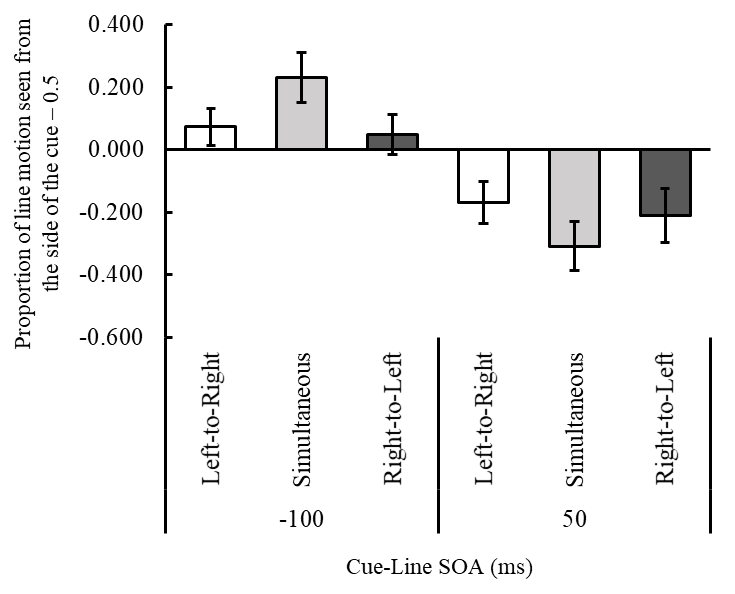


Figure A2. The proportion of ILM perceived at each SOA between the cue and line in each presentation direction of the line in Experiment 2. Positive values on the y-axis indicate that line motion was perceived from the cue side and negative values indicate that it was toward the cue side. Error bars indicate 95% confidence intervals. The responses to line motion in three directions (left to right, simultaneous, or right to left) were analyzed separately. First, responses to left-to-right line motion were included in the analysis. A one-sample *t*-test to determine whether the ILM was greater or less than zero at each SOA was performed, and the results demonstrated that when the SOA was –100 ms, the ILM was significantly greater than 0 (*t*[18] = 2.590, *p* = .018, Cohen’s *dz* = 0.594); conversely, when the SOA was 50 ms, the ILM was significantly smaller than zero (*t*[18] = 5.295, *p* < .001, Cohen’s *dz* = 1.214). Next, responses to no line motion (simultaneous) were included in the analysis. The results of a one-sample *t*-test demonstrated that when the SOA was –100 ms, the ILM was significantly greater than 0 (*t*[18] = 6.020, *p* < .001, Cohen’s *dz* = 1.381); conversely, when the SOA was 50 ms, the ILM was significantly smaller than zero (*t*[18] = 8.250, *p* < .001, Cohen’s *dz* = 1.893). Finally, responses to right-to-left line motion were included in the analysis. The results of a one-sample *t*-test demonstrated that when the SOA was –100 ms, the ILM was not significantly different from 0 (*t*[18] = 1.648, *p* = .117, Cohen’s *dz* = 0.378). When the SOA was 50 ms, the ILM was significantly smaller than zero (*t*[18] = 5.121, *p* < .001, Cohen’s *dz* = 1.175).


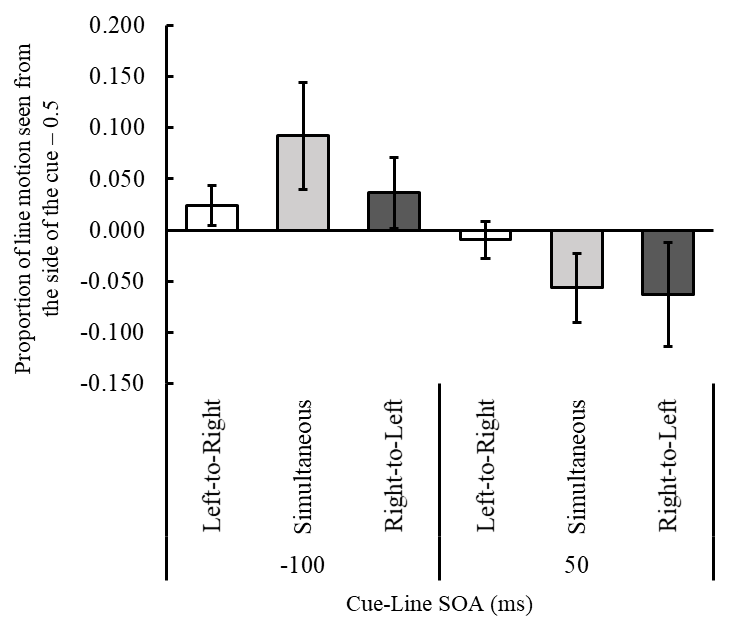


Figure A3. The proportion of ILM perceived at each SOA between the cue and line in each presentation direction of the line in Experiment 3. Positive values on the y-axis indicate that line motion was perceived from the cue side and negative values indicate that it was toward the cue side. Error bars indicate 95% confidence intervals. The responses to line motion in three directions (left to right, simultaneous, or right to left) were analyzed separately. First, responses to left-to-right line motion were included in the analysis. A one-sample *t*-test to determine whether the ILM was greater or less than zero at each SOA was performed, and the results demonstrated that when the SOA was –100 ms, the ILM was significantly greater than 0 (*t*[18] = 2.535, *p* = .020, Cohen’s *dz* = 0.567). When the SOA was 50 ms, the ILM was not significantly different from 0 (*t*[18] = 1.102, *p* = .284, Cohen’s *dz* = 0.246). Next, responses to no line motion (simultaneous) were included in the analysis. The results of a one-sample *t*-test demonstrated that when the SOA was –100 ms, the ILM was significantly greater than 0 (*t*[18] = 3.680, *p* = .002, Cohen’s *dz* = 0.823); conversely, when the SOA was 50 ms, the ILM was significantly smaller than zero (*t*[18] = 3.469, *p* = .003, Cohen’s *dz* = 0.776). Finally, responses to right-to-left line motion were included in the analysis. The results of a one-sample *t*-test demonstrated that when the SOA was –100 ms, the ILM was significantly greater than 0 (*t*[18] = 2.595, *p* = .018, Cohen’s *dz* = 0.495); conversely, when the SOA was 50 ms, the ILM was significantly smaller than zero (*t*[18] = 2.595, *p* = .018, Cohen’s *dz* = 0.580).


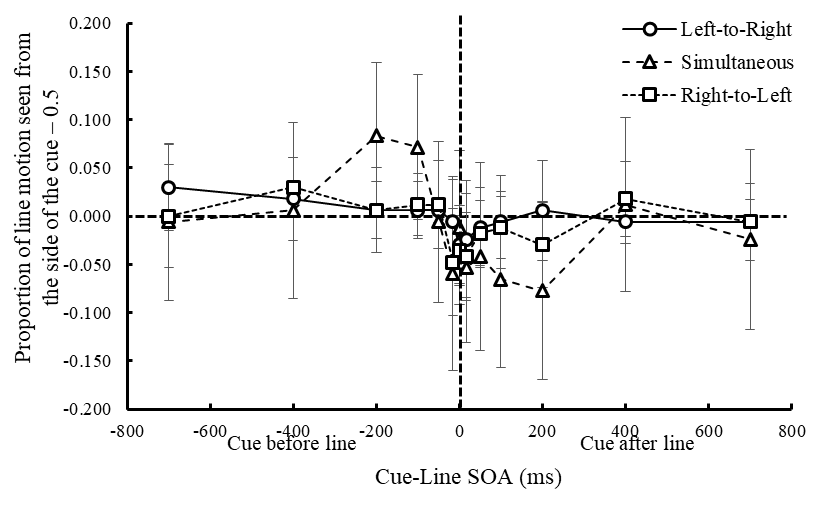


Figure A4. The proportion of ILM perceived at each SOA between the cue and line in each presentation direction of the line in Experiment 4. Positive values on the y-axis indicate that line motion was perceived from the side of the cue and negative values indicate that it was toward the cue side. Error bars indicate 95% confidence intervals. The responses to line motion in three directions (left to right, simultaneous, or right to left) were analyzed separately.

First, responses to left-to-right line motion were included in the analysis. A two-way ANOVA with two within-subject variables on the outcome variable showed no significant main effect of the cue location (*F*[1, 13] = 0.112, *p* = .743, *η_p_*^2^ = .009). The main effect of SOA was significant (*F*[12, 156] = 2.247, *p* = .012, *η_p_*^2^ = .147). The interaction was not significant (*F*[12, 156] = 0.651, *p* = .795, *η_p_*^2^ = .048). Next, responses to no line motion (simultaneous) were included in the analysis. A two-way ANOVA showed no significant main effect of the cue location (*F*[1, 13] = 1.313, *p* = .273, *η_p_*^2^ = .092). The main effect of SOA was significant (*F*[12, 156] = 2.185, *p* = .015, *η_p_*^2^ = .144). The interaction was not significant (*F*[12, 156] = 1.503, *p* = .128, *η_p_*^2^ = .104). Finally, responses to right-to-left line motion were included in the analysis. A two-way ANOVA showed no significant main effects of the cue location (*F*[1, 13] = 2.721, *p* = .123, *η_p_*^2^ = .173) and SOA (*F*[12, 156] = 1.249, *p* = .255, *η_p_*^2^ = .088). The interaction was not significant (*F*[12, 156] = 1.797, *p* = .053, *η_p_*^2^ = .121).


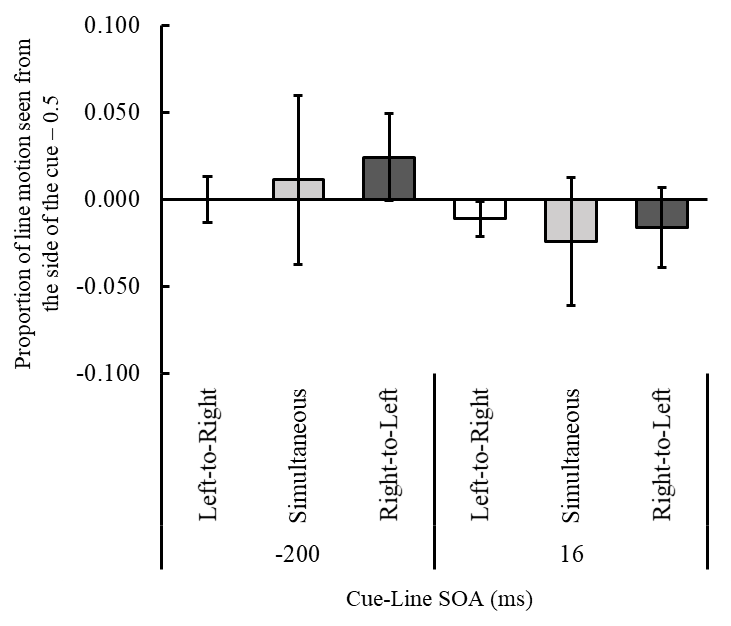


Figure A5. The proportion of ILM perceived at each SOA between the cue and line in each presentation direction of the line in Experiment 5. Positive values on the y-axis indicate that line motion was perceived from the cue side and negative values indicate that it was toward the cue side. Error bars indicate 95% confidence intervals. The responses to line motion in three directions (left to right, simultaneous, or right to left) were analyzed separately. First, responses to left-to-right line motion were included in the analysis. A one-sample *t*-test to determine whether the ILM was greater or less than zero at each SOA was performed, and the results demonstrated that when the SOA was –100 ms, the ILM was not significantly different from 0 (*t*[30] = 0.000, *p* = 1.000, Cohen’s *dz* = 0.000). When the SOA was 50 ms, the ILM was significantly smaller than zero (*t*[30] = 2.224, *p* = .032, Cohen’s *dz* = 0.403). Next, responses to no line motion (simultaneous) were included in the analysis. The results of a one-sample *t*-test demonstrated that when the SOA was –100 ms, the ILM was not significantly different from 0 (*t*[30] = 0.474, *p* = .639, Cohen’s *dz* = 0.085). When the SOA was 50 ms, the ILM was not significantly different from 0 (*t*[30] = 1.337, *p* = .191, Cohen’s *dz* = 0.240). Finally, responses to right-to-left line motion were included in the analysis. The results of a one-sample *t*-test demonstrated that when the SOA was –100 ms, the ILM was not significantly different from 0 (*t*[30] = 1.976, *p* = .057, Cohen’s *dz* = 0.355). When the SOA was 50 ms, the ILM was not significantly different from 0 (*t*[30] = 1.437, *p* = .161, Cohen’s *dz* = 0.258).
